# Recent progress in adsorptive removal of different contaminants by chitosan-based aerogel

**DOI:** 10.1039/d5ra03536h

**Published:** 2025-08-22

**Authors:** Mohammed Ahmed Shehab, Munaf Al-lami, Mohammad A. Taher, Haidar Hasan Mohammed, Adnan A. AbdulRazak, Khalid T. Rashid, Alhafadhi Mahmood, Mohammed Faleh Abd Al-Ogaili, Saad Alsarayefi

**Affiliations:** a Polymers and Petrochemicals Engineering Department, Basrah University for Oil and Gas Basrah 61004 Iraq mohammed.ahmed@buog.edu.iq; b Department of Chemical Engineering and Petroleum Refining, Basrah University for Oil and Gas Basrah 61004 Iraq munaf.adnan@buog.edu.iq moh.may@buog.edu.iq haidar.alawaad@buog.edu.iq; c Thermodynamics and Mathematical Physics Unit, University of Mons 7000 Mons Belgium; d Department of Chemical Engineering-University of Technology Baghdad Iraq adnan.a.alsalim@uotechnology.edu.iq Khalid.t.rashid@uotechnology.edu.iq; e Department of Mechanical and Energetic, University of Dunaújváros Dunaújváros Hungary Alhafadhi@uniduna.hu; f Department of Mechanical Engineering, University of Sumer Thi-Qar Iraq mahmoodhs199@gmail.com; g Department of Aeronautical Engineering Technologies, Alfarahidi University Baghdad Iraq m.falehabd@uoalfarahidi.edu.iq; h Petroleum & Gas Engineering Department, University of Thi-Qar 64001 Nasiriyah Iraq saad.j.n@utq.edu.iq

## Abstract

Rapid industrial development has led to the discharge of significant amounts of untreated industrial wastewater into the environment, resulting in substantial effects on natural ecosystems and human health. Consequently, there is a need to develop new environmentally friendly alternatives for water remediation. In this regard, chitosan (CS) aerogels possess high porosity, low density, and biodegradability, and act as effective sorbents for the removal of various ionic pollutants from water, air, and soil. The presence of numerous amino (–NH_2_) and hydroxyl (OH) groups enhances the adsorption of ionic pollutants *via* electrostatic interactions, hydrogen bonding, and chelation mechanisms. This review provides an overview of the use of chitosan-based aerogels as eco-friendly gels to remove different contaminants from water such as dyes, heavy metals, microorganisms, and pharmaceuticals, by improving the mechanical strength, decreasing hydrophilicity, and increasing acid stability. These improvements are accomplished using different modification methods, such as blending with nanofillers, chemical and/or ionic crosslinkers, and designing composite aerogels. The modified aerogel exhibits excellent adsorption ability against different contaminants, as well as antibacterial properties, making it promising candidates for a wide range of applications.

## Abstract 2

Preparation steps of chitosan.

## Graphical abstract

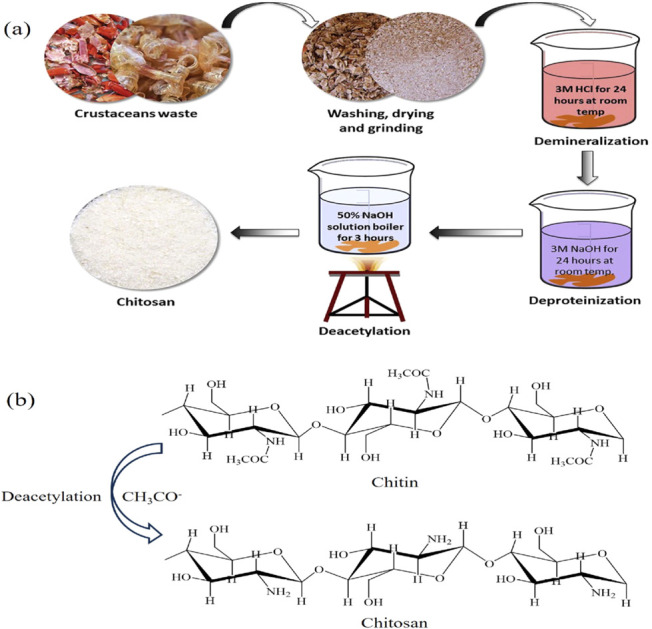

## Introduction

The global scarcity of water resources has escalated owing to the growing disparity between freshwater supply and consumption. The growing population and migration to drought-affected regions, driven by rapid industrial advancement, heightened per capita water consumption, and climate change, have resulted in altered weather patterns in urban areas.^1^ Various known and emerging pollutants such as organic dyes, heavy metals, antibiotics, pesticides, and microorganisms have been detected in natural water bodies.^2^ Numerous efforts have been made by scientists and researchers to eliminate these pollutants from water using various decontamination processes. These processes involve physical, biological, and chemical methods. The technologies can be categorized into three groups: conventional methods, which include activated sludge^3^ coagulation–flocculation^4^ chemical precipitation^5^ adsorption^6,7^ and filtration;^8^ established recovery methods, such as ion exchange^9^ solvent extraction,^10^ electrochemical treatments,^11^ and membrane bioreactors;^12^ and emerging methods, which include advanced oxidation,^13^ biosorption,^14^ adsorption into non-conventional materials,^15^ and nanofiltration.^16^ Many biological and chemical processes exhibit low productivity, slow kinetics, limited scalability, and a tendency to produce hazardous intermediates.^17^ In this context, wastewater treatment faces multiple challenges concerning the presence of emerging pollutants, *i.e.*, organic dyes, heavy metals, microorganisms, fertilizers, and herbicides, which are not typically addressed by conventional treatment methods.^18^ Among them, adsorption is a prevalent and efficient technique for wastewater treatment due to its convenience, simplicity, cost-effectiveness, and absence of harmful by-products.^19^ Generally, adsorption effectiveness depends on the interaction between the adsorbent and adsorbate; specifically, the adsorbent must be selected based on the particular molecule targeted for removal from the polluted solution. Furthermore, adsorption occurs at the solid–liquid interface; hence, porous materials are highly preferable.^20^ Many kinds of adsorbents are used for the removal of water pollutants, such as clay,^21^ carbon materials,^22,23^ and metal oxides.^24,25^ Some of them have drawbacks such as limited adsorption capacity, inadequate recyclability, and selectivity.^26^ Furthermore, the prospective utilization of these materials in large-scale water treatment raises concerns about the incorporation of hazardous chemicals in their synthesis and the leaching of these materials during the adsorption process. To overcome this, alternatives including the utilization of green, natural materials and their incorporation into biopolymer aerogels have drawn more attention over the past decade. Green and natural adsorbents are obtained from renewable resources and produced through eco-friendly methods.^27^ These materials, including cotton fiber, wood, biopolymer, and zeolite, are extensively utilized as adsorbents for decontamination processes due to their significant specific surface area, low cost, availability, porous structures, and environmentally friendly.^28^

**Fig. 1:**
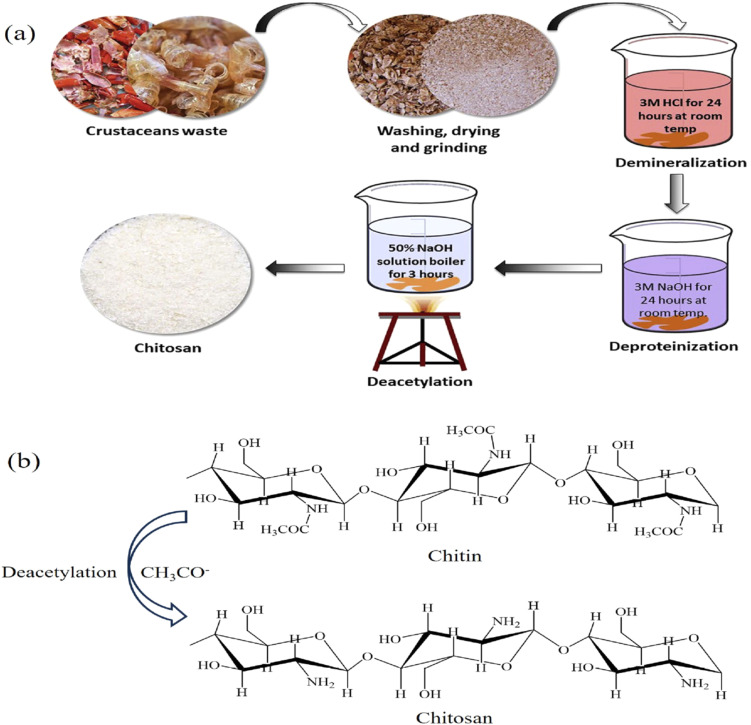
(a) Schematic diagram for the preparation steps of chitosan. (b) Deacetylation of chitin.^35^

One of the most important abundant natural biopolymers, chitosan (CS), has received attention in wastewater treatment due to various factors, such as ecologically sustainable, biodegradable, biocompatible, non-toxic, and economical material.^29,30^ Chitosan is a linear polysaccharide with a structure abundant in functional groups, including amino and hydroxyl groups generally derived from chitin, as illustrated in Fig. 1. Chitin is extracted from the source by a series of procedures. Initially, the raw materials undergo treatment with 3 M hydrochloric acid (HCl) to facilitate demineralization. This technique eliminates mineral constituents in the form of inorganic salts (ashes) while preserving the chitin structure and preventing depolymerization. In the subsequent stage, proteins are eliminated using a dilute base (NaOH solution). The chitin is subsequently extracted from the reaction mixture by eliminating other organic components. Then, chitin undergoes alkaline deacetylation to be transformed into chitosan, during which acetamido groups are changed into amino groups. Deacetylation occurs in highly alkaline conditions (50% NaOH solution) and at elevated temperatures (80–120 °C). Finally, chitosan is dried to get an optimal moisture content in the final product.^31,32^

Despite the significant advantages of chitosan (CS), its direct application in certain systems is limited by its relatively low absorption capacity in neutral solvents. This is primarily due to strong inter- and intramolecular hydrogen bonding, which reduces the availability of polar functional groups (such as –OH and –NH_2_) for interaction with water molecules. These hydrogen bonds also decrease the free volume between polymer chains, limiting water penetration and swelling.^33^ To tackle this issue, CS-based three-dimensional aerogel preparation is the common method for improving adsorption performance.^34^

**Fig. 2:**
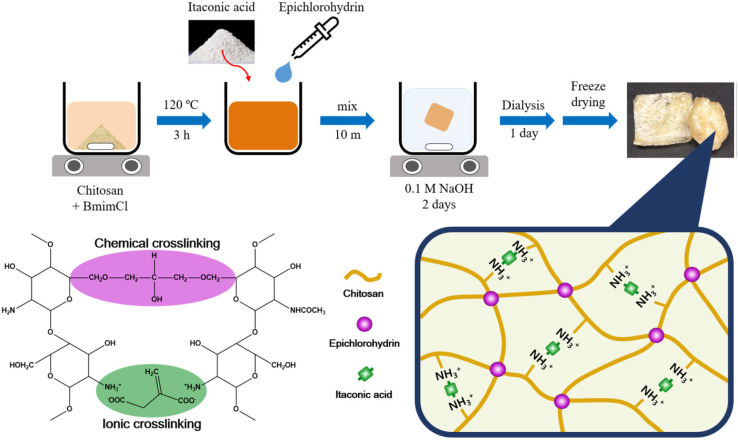
Schematic diagram of dual-crosslinked chitosan aerogel preparation steps.^40^

Aerogel, a three-dimensional solid developed through the physical or chemical cross-linking of polymer chains followed by the drying of gels using various techniques (*e.g.*, supercritical drying or freeze-drying), demonstrates exceptional efficacy in the removal of dyes and metal ions owing to its high specific surface area, porosity exceeding 80.00%, and low densities ranging from 0.005 to 0.5 g cm^−3^, in addition to its superior capacity for separation and recycling from aqueous solutions while maintaining structural integrity.^36–39^Fig. 2 illustrates the preparation procedures for chitosan aerogel, which use epichlorohydrin (ECH) and itaconic acid (IA) as chemical and ionic crosslinkers. Chitosan is dissolved in 1-butyl-3-methylimidazolium chloride (BmimCl) solvent and stirred for 3 hours at 120 °C, followed by mixing with ECH. For dual crosslinking, IA was included; the solutions were immersed and agitated in a 0.1 M NaOH solution at ambient temperature for 48 hours. Subsequent to dialysis, the hydrogels were rinsed with distilled water to eliminate unreacted ECH. Ultimately, the hydrogels underwent freeze-drying for 24 hours at – 95 °C, while the aerogels were maintained in a vacuum oven overnight.^40^ To the best of our knowledge, many reviews have studied the preparation, characterization, and application of chitosan aerogel. However, the adsorption abilities of chitosan aerogel as an eco-friendly gel toward various types of contaminants, such as dyes, heavy metals, microorganisms, and pharmaceuticals, have not been extensively reviewed.

## CS-based aerogel for water decontamination

CS is being used widely as an adsorbent because of remarkable properties as explained earlier. The amino (–NH_2_) and hydroxyl (–OH) groups in CS facilitate chemical interactions and crosslinking, which contribute to the formation of robust, acid-stable films and interactions *via* hydrogen bonding and van der Waals forces. These properties make CS an exceptional adsorbent for dyes and various contaminants in wastewater treatment.^32,41–43^ In the following sections, we will explain the adsorption ability of CS-based aerogels against different water contaminants, such as dyes, heavy metals, pharmaceuticals waste, microorganisms and their removal mechanisms.

### Removal of dyes

The textile products have increased by about 7 × 10^5^ tons per year worldwide, and due to discharged untreated dye effluents (approximately 2.8 × 10^5^ tons) to the water bodies, causing water contamination, which has posed many serious threats to the ecological system, plant growth, and human health.^44^ Furthermore, these dyes are poisonous, hydrophilic, mutagenic, resistant to degradation, and reduce the rate of photosynthesis and the concentration of dissolved oxygen in aquatic environments.^45^ In this regard, CS is abundant in amine and hydroxyl groups, enabling it to interact with anionic and cationic dyes. Anionic dyes can attach to the amino group due to the electrostatic attraction between the positive charge on the amino groups and the negative charge on the dye's molecules.^46^ However, chitosan aerogels have some drawbacks, such as low mechanical strength, water stability and high hydrophilicity, which hinder their interactions with hydrophobic organic contaminants.^47,48^ To overcome this issue, CS-based aerogel should be combined with other materials. For instance, Varamesh and his co-worker developed composite biobased aerogel containing CS, cellulose filament (CF), and citric acid (CA) for methylene blue (cationic dye 50 mg L^−1^) removal. The results demonstrated that the composite-based aerogel has high mechanical properties (up to ∼65 kPa m^3^ kg^−1^) and high dye adsorption removal (619 mg g^−1^, 99%, pH = 6) due to electrostatic interactions between CF and CS leading to enhanced diversity of functional groups (carboxylic acids, hydroxyls, and amines) on the surface of the materials. In addition, CA acts as a covalent crosslinker.^49^ Tan *et al.* synthesized a CS and quinoa polysaccharide (QS) composite aerogel and investigated the adsorption capacity toward two types of dyes, Congo red (CR, 50 mg L^−1^) and methylene blue (MB, 5 mg L^−1^), which are anionic and cationic, respectively. They found the maximum adsorption capacity for CR was 342 mg g^−1^ at pH = 6. It could be attributed to the zeta potential of the composite being positive when pH increased from 4 to 6 while decreased with pH increase from 6 to 12. On the other hand, the adsorption capacity for MB was 57.80 mg g^−1^ at pH = 12. Because of the low pH value of the solution likely caused a high concentration of H^+^ ions surrounded the adsorption site, which prevented the dye's approach. Furthermore, at lower pH levels, the amino group in the CR structure became protonated and subsequently polymerized with the shell. The protonated amino groups and hydroxyl groups on the sugar molecule chain produce electrostatic repulsion. As the pH value rises, the repulsive force decreases, leading to an increased adsorption capacity. However, with further increases in pH, the gravitational force between the aerogel and CR also decreases, resulting in a decrease in adsorption capacity.^50^ Some scientists' efforts tried to use by-product waste to improve the adsorption ability of CS-based aerogel; in this regard, Martina and Mariano prepared composite aerogel made from CS and soot to enhance the adsorption ability toward cationic and anionic dyes such as MB and indigo carmine (IC), respectively. They concluded that the adsorption properties of modified CS-based aerogel against MB were higher than pristine CS-based aerogel due to electrostatic interactions between amino groups and MB molecules, whereas pristine CS-based aerogel had a higher adsorption ability toward IC than CS/soot aerogel; this could be attributed to the interaction between soot particles and CS blocking the CS active sites.^51^ As mentioned earlier, one of the drawbacks of chitosan (CS) is its hydrophilicity, which negatively affects its mechanical strength. To address this issue, Natália *et al.* prepared highly hydrophobic composite aerogel containing CS beads, cellulose nanocrystals (CNC), and hydrophobized tannic acid (HTA) and tested the adsorption performance for removing basic blue 26 and basic brown 4 dyes in batch and fixed-bed assays. In batch experiments, the results show that the CS@CNC-HTA beads aerogel has higher adsorption removal of 375.58 mg g^−1^ and 235.39 mg g^−1^ than pure CS, 330.59 mg g^−1^ and 213.74 mg g^−1^ for basic blue 26 and basic brown 4, respectively. Meanwhile, in fixed bed experiments, the CS@CNC-HTA beads aerogel has efficiently removed basic blue 26.^52^ In another study, the impact of functionality on the adsorption capacity of CS-based aerogel has been studied by Zhang and his colleagues. They prepared ferrocene (Fc)-functionalized CS-based aerogels and examined their adsorption ability against MB with and without H_2_O_2_. The results demonstrated that the adsorption performance of Fc/CS-based aerogel without using H_2_O_2_ was low (<40 mg g^−1^, 8.2%), whereas it was 9.29% for pristine CS aerogel; on the other hand, the adsorption efficiency for Fc/CS-based aerogel in the presence of H_2_O_2_ was 92.79% in comparison with pristine CS aerogel (19%).^53^ Another drawback to limiting the use of CS-based aerogels in some applications is the dense structure, which may limit the adsorption capacity. To solve this problem, Kuang *et al.* developed a 3D hierarchically macro–*meso*–microporous structure with a high-strength chitin/chitosan-based composite aerogel (HPS-aerogel) and investigated the adsorption ability to remove CR (500 mg L^−1^). The HPS-aerogel has high adsorption capacity (2074 mg g^−1^) at pH = 6.5. Because under acidic conditions, the amino groups are protonated, making H^+^ ions occupy the active sites on the aerogel, causing low absorbability for CR, while, at alkaline conditions, the amino groups are deprotonated, making OH^−^ ions occupy the active sites, leading to decreased electrostatic interactions. The maximum adsorption ability is attributed to the high porosity of the 3D aerogel structure.^54^ Marotta *et al.* synthesized a zeolite/chitosan (CS) aerogel and assessed its adsorption efficacy for cationic (methylene blue, MB) and anionic (indigo carmine, IC) dyes. The maximal adsorption capabilities were determined to be 221 mg g^−1^ for IC and 108 mg g^−1^ for MB. The significant adsorption was ascribed to the abundant active sites in the microporous structure of the aerogel, which enabled fast diffusion and effective pollutant elimination.^55^ Other studies have used CS-based composite aerogel as an eco-friendly gel to remove different types of cationic and anionic dyes, as shown in Table 1.

**Table 1:** Different CS-based aerogels for dyes adsorption^a^

Aerogel	Dye	Initial concentration mg L^−1^	pH	Adsorption capacity mg g^−1^	Removal %	References
CS/AG/MC	MB	50–400	9.1	73.1	87.5	56
	Acid black-172		4.85	71.5	82	
SMCS	MO	50	—	57.65	—	57
	MB			51.62		
	RhB			58.65		
	Sudan I			48.37		
CS/CNF	AB93	175	2	1428.7	∼99	58
BNCC/CMCT	MB	240	7.5	785	83.5	59
β-CD-CS@HMTA	MB	500	2	395.7	—	60
	RhB			364.3		
	Alizarin red S			261		
	Acid orange 7			134.1		
NBNC/CS	Bromophenol blue	4000	2–9	29.842 g g^−1^	—	61
	Direct blue 6			20.927 g g^−1^		
CS-PDA	Plastic	500	7.45	118.7	—	62
	MB	1000	6.35	734.4		
	Alizarin red S	500	4.43	288.8		
	Acid orange 7	500	6.72	214.3		
	RhB	1000	5.15	495.3		
CS/Zeolite	MB	750	—	108	92	55
	IC			221		
TEMPO-C/CS	MO	300	6	136.64	91.82	63
	MB	25	7	31.56	47	
DCBA bead	MB	500	10	653.3	91.4	64
	CR	2300	6	559.6	94.7	

a CS: chitosan; PDA: polydopamine; β-CD: β-cyclodextrin; HMTA: hexamethylenetetramine; BNCC: bifunctional nanocrystalline cellulose; CMCT: carboxymethylated chitosan; CNF: cellulose nanofibril; AG: alginate; DCBA: double-cross-linked biohybrid; MC: modified clay composite; SM: sulfhydryl modified; NB: nanobentonite; NC: nanocellulose; TEMPO: 2,2,6,6-tetramethylpiperidinyloxy; C: cellulose; MO: methyl orange; RhB: rhodamine B; AB93: acid blue 93.

### Dyes adsorption removal mechanism

**Fig. 3:**
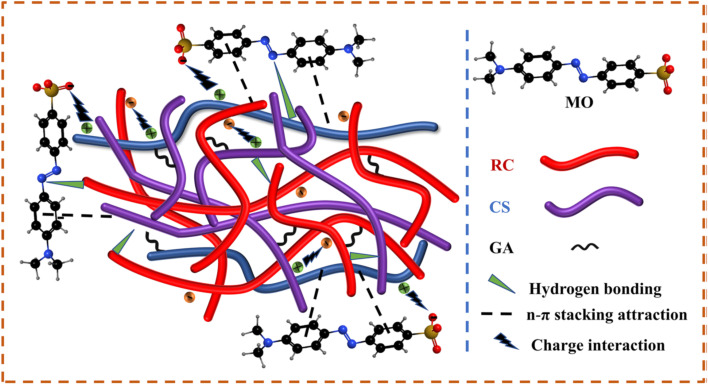
Adsorption mechanism of methyl orange (MO) using regenerated cellulose/chitosan composite aerogel (RC/CSGA).^65^

The adsorption ability of CS-based different composite aerogels as an eco-friendly gel for removing different types of dyes has been explained earlier. Now, we need to address a crucial question: how do these aerogels remove dyes from water? Dyes ions can exist either cationic (positive charge) or anionic (negative charge) and each type has a special mechanism. In general, the adsorption mechanism responsible for removing these dyes involves electrostatic attraction, hydrogen bonding, and n–π interaction. He *et al.* presumed the adsorption mechanism between the (MO anionic dye) and regenerated cellulose/chitosan composite aerogel (RC/CSGA) using glutaraldehyde (GA) as the crosslinking agent as follows: in acidic conditions; the amino group of aerogels is protonated, producing (–NH_3_^+^ and –NH_2_^+^) cationic groups with positive charges on the aerogel surface and the negative charge of the sulfonate group (–SO_3_^−^) on MO dye, leading to strong electrostatic attraction. Furthermore, hydrogen bonds formed between hydrogen on the aerogel surface and oxygen/nitrogen in MO, the adsorption mechanism illustrated in Fig. 3.^65^ While, in alkaline conditions, the amino groups on the aerogel surface are deprotonated, changing –NH_3_^+^ into neutral –NH_2_ which reduces the surface positive charge and may enhance the adsorption capability with cationic dyes *via* hydrogen bonding or other interactions.

### Removal of heavy metals

Numerous industrial and human activities, including industrial facilities, transportation, metallurgy, wastewater, combustion processes, mining, and agriculture, are the principal contributors to the release of heavy metals into aquatic ecosystems.^66^ Heavy metals such as chromium, copper, nickel, cadmium, zinc, mercury, lead, *etc.*, existing in water are a critical problem that should be handled effectively due to the substantial threat they pose to the sustainability of ecosystems and human health.^67,68^ The non-biodegradability, toxicity, and ability to accumulate in the human body present significant issues, posing long-term risks to human health and natural ecosystems.^69^ Researchers have explored and tried many technologies, such as ion-exchange, electrolysis, flocculation, and chemical precipitation, to mitigate the risks posed by heavy metals in aqueous solutions.^70–73^ Using these processes, a significant amount of contaminated water has been partially remediated. Nevertheless, the majority of these approaches are accompanied by drawbacks, including secondary pollutants, high costs, and low effectiveness, which restrict their wide use.^74^ In contrast, bio-adsorption has been demonstrated to be the most suitable approach due to its operational flexibility, cost-effectiveness, and absence of secondary pollutants.^75^ For instance, Wang and Li developed a three-dimensional (3D) interconnected porous chitosan aerogel that incorporates a combination of chitosan gelation and soft fine bubbles. The templates were initially employed using ultrasonic technology, with natural genipin, derived from the hydrolysis of geniposide by β-glucosidase, selected as the crosslinker. After that they investigated the adsorption ability toward copper ion (Cu^2+^) and the results demonstrated that the aerogel has excellent adsorption performance with an adsorption capacity of 116.7 mg g^−1^. The existence of numerous interconnected pores facilitates the exposure of active adsorption sites, resulting in enhanced adsorption efficiency.^76^ Tan *et al.* successfully prepared pineapple leaves and chitosan composite aerogels with different drying techniques. The as-prepared aerogels were highly porous, had strong acid resistance and were ultra-lightweight. The adsorption capacity has been examined to remove Cr(vi) from water, and they found that the drying technique does not affect the adsorption capacity, whereas the optimum pH was 3 with 211.4 mg g^−1^ of high adsorption of Cr(vi).^77^ To protect the environment not only by eliminating the pollutants but also by using waste materials, Li and his co-workers prepared aerogel by combining chitosan and waste office paper to remove (Cu^2+^). They have aerogels with acid resistance, excellent mechanical strength, and high adsorption capacity (156.3 mg g^−1^).^78^ In another work, Li *et al.* modified chitosan with ethylenediamine tetraacetic anhydride to improve adsorption ability toward different types of heavy metals such as Cu^2+^, Pb^2+^, and Cd^2+^. The adsorption capacity at pH = 5 was 108.14, 143.73 and 84.62 mg g^−1^ for Cu^2+^, Pb^2+^, and Cd^2+^, respectively.^79^ To investigate the adsorption capacity of CS-based aerogels against different heavy metals, Shahnaz *et al.* prepared a composite aerogel containing nanobentonite-incorporated nanocellulose/chitosan aerogel (NCNB) and examined its adsorption capacity for Cr(vi), Co(iii), and Cu(ii) at different pH levels. They found that the maximum removal efficiency was 98.9% (Cr(vi)), 97.45% (Co(iii)), and 99.01% (Cu(ii)) at optimum pH levels of 4, 2, and 5, respectively.^80^ As mentioned before, Cs-based aerogels have many features, among them high porosity, and to explore their relevance in adsorptive removal of heavy metals, Li *et al.* developed a cross-linked aerogel as an environmentally sustainable adsorbent characterized by low density (0.0283 g cm^−3^) and high porosity (97.98%) and examined its adsorption capability for copper ions. The findings indicated that the synthesized aerogel demonstrated an exceptional adsorption capacity of 21.38 mg g^−1^. They concluded that the adsorption mechanism was facilitated by the aerogel's high porosity, which enabled liquid absorption *via* capillary forces produced by the porous structure and reinforced by hydrogen bonding.^81^ In the same context, Vareda *et al.* synthesized CS/silica aerogel with (96% porosity, 17 cm^3^ g^−1^ pore volume, 33 μm pore diameter and 2.05 m^2^ g^−1^ specific surface area) and evaluated its adsorption capacity for copper ions. The results showed that the maximum adsorption capacity of 40 mg g^−1^ and they also observed decrease in porosity because of the interaction between aerogel and metal ions as well as the pore filling.^82^ Many studies investigate the adsorption performance of CS-based different composite aerogels to remove different types of heavy metals, as summarised in Table 2.

**Table 2:** Different CS-based aerogels for heavy metals adsorption^a^

Aerogel	Heavy metal	Initial concentration mg L^−1^	pH	Adsorption capacity mg g^−1^	Removal %	References
CS/silica	Cd(ii)	200	3	64.74	96.84	83
CS/NFC	Pb(ii)	150	—	252.6	85	84
AG/ME/CS	Pb(ii)	2000	5.5	1331.6	95	85
CS/AG/MC	Cr(vi)	400	3.1	62.4	75	56
SM/CS	Cu(ii)	200	6	81.15	—	57
	Pb(ii)		5	38.87		
	Cd(ii)		6	38.15		
β-CD-CS@HMTA	Cr(vi)	100	2	333.8	—	60
CS-PDA	Cr(vi)	100	2	374.4	80	62
	Pb(ii)		5.5	441.2		
PDA@CNT/CS	As(v)	150	7	39	93.6	86
HPS	Cu(ii)	200	5	59.21	—	54
PCCSA	Cu(ii)	240	6	175.56	96.9	87

a NFC: nanofibrillated cellulose; PDA: polydopamine; CS: chitosan; CNT: carbon nanotube; β-CD: β-cyclodextrin; HMTA: hexamethylenetetramine; AG: alginate; ME: melamine; HPS: high-strength chitosan; MC: modified clay composite; PCCSA: polyethyleneimine-modified carboxymethyl chitosan aerogel; SM: sulfhydryl modified.

### Heavy metals adsorption removal mechanism

**Fig. 4:**
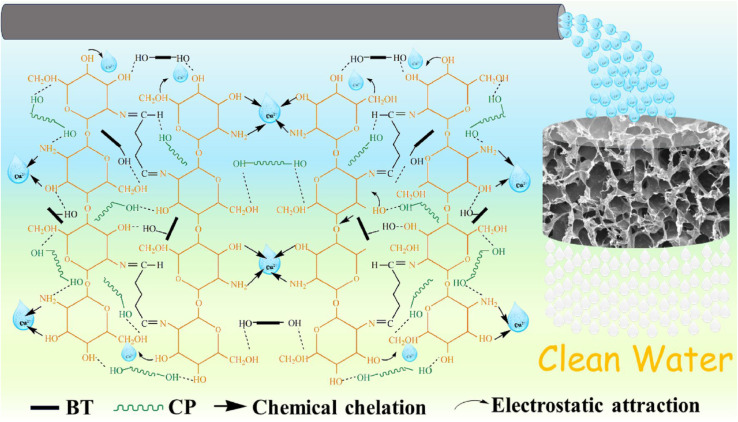
Suggested adsorption mechanism of copper ions (Cu^2+^) using citrus peel/chitosan/bentonite (CP/CS/BT) aerogel.^87^

Chitosan-based aerogels possess a significant number of amino and oxygen-containing functional groups (hydroxyl and carbonyl groups) on their surface, which facilitates the adsorption of different heavy metal ions. To clarify the importance of these functional groups through the adsorption process, Nie and his colleagues used citrus peel (CP), chitosan (CS), and bentonite (BT) to prepare a 3D porous aerogel for the removal of Cu(ii) ions from wastewater. The results showed that the aerogel has an impressive adsorption capacity of 861.58 mg g^−1^ at pH = 5.5, and they proposed the adsorption mechanism (shown in Fig. 4) to be based on electrostatic attraction and chemical chelation. BT in the aerogel provides a negative surface charge, which attracts the positively charged Cu(ii) ions electrostatically. Furthermore, –NH_2_ and –OH groups act as bidentate or tetradentate chelate ligands with copper ions.^88,89^

### Removal of microorganisms

The WHO states that infectious diseases are induced by harmful microorganisms, including bacteria, viruses, parasites, or fungi. These infectious diseases can be transmitted directly or indirectly between individuals. Zoonotic diseases are infectious ailments originating in animals that can be transmitted to humans, resulting in illness,^90^ make them seriously threatening to human health, living organisms, and the environment. Much effort focuses on removing these threats; however, it is still a challenge due to some limitations, such as high cost, limited efficiency, and harmful byproducts. As mentioned before, biopolymer has remarkable features; among them are low cost and no intermediate product. Chitosan possesses many amino (–NH_2_) and hydroxyl (–OH) groups, which can act as sites of coordination to form complexes with pollutant ions.^91^ Nevertheless, CS alone is inadequate owing to its weak chemisorption and limited antimicrobial efficacy. Consequently, it is extremely important to develop a scalable and eco-friendly approach to transform CS into multifunctional material exhibiting superior performance across various fields,^92^ hence, chitosan aerogel, with the aforementioned advantages, demonstrates significant potential for the effective removal of various microorganisms. Nonetheless, limitations persist, including the low mechanical strength and high hydrophilicity of pure chitosan aerogel. To address these obstacles, an effective approach is the incorporation of appropriate fillers into the chitosan matrix.^93^ Ko and Kim prepared dual crosslinked chitosan aerogel using itaconic acid (IA) and epichlorohydrin (ECH) as ionic and chemical crosslinking, respectively, and evaluated the antibacterial performance against Gram-negative bacteria (*E. coli*) and Gram-positive bacteria (*C. glutamicum*). The results show that the dual crosslinked aerogel has excellent antibacterial properties against both bacteria. It is attributed to the positive charge of NH_3_^+^ groups on aerogel electrostatic interaction with the negative charge cell of bacteria, leading to destroying it ref. 40. Tang *et al.* prepared a multifunctional biomass composite aerogel containing chitosan (CS), nanocellulose (NC), and sodium phytate (PA-Na) and investigated its antibacterial properties against Gram-positive *S. aureus* and Gram-negative *E. coli.* The results demonstrated that the composite aerogel has good antimicrobial activity with the removal rate of 78.5% for *S. aureus* and 89.2% for *E. coli*. They proposed the removal mechanism based on the zeta potential of composite aerogel with a high positive charge that could adhere to the bacteria surface by electrostatic attraction, causing the destruction of the membrane cell and then cell death.^94^ Julia and her co-workers synthesized chitosan-based aerogel modified with Tiliaplatyphyllos extract as a crosslinker and evaluated the antibacterial properties against *S. aureus* (Gram-positive bacteria). They observed that the modified aerogel has good antibacterial activity due to electrostatic attraction between the NH_3_^+^ on the aerogel surface and the negative charge of lipoteichoic acids on the bacterial cell membrane, which causes blocking nutrient diffusion to the cell.^95^ In other work, Rizal *et al.* fabricated cellulose nanofibers (CNFs) and chitosan (CS) aerogel and examined the antibacterial ability towards Gram-negative and Gram-positive bacteria with initial bacteria counts of 1.34 × 10^8^ CFU mL^−1^ and 1.56 × 10^8^ CFU mL^−1^ for *E. coli* and *S. aureus*, respectively. They found the aerogel showed a 2-log reduction (99%) against both bacteria.^96^ Similarly, Zhang and his colleagues investigated the antibacterial performance of as-prepared chitosan-based aerogel embedded amino-functionalized molybdenum disulfide nanosheets (CS/NMNSs) against *E. coli* and *S. aureus* and concluded the inactivation rate of bacteria for *E. coli* was 97.52% and for *S. aureus was* 99%. Gram-positive bacteria have significantly lower isoelectric points than Gram-negative bacteria, resulting in a considerably larger negative charge at the same pH level. Chitosan is positively charged, resulting in a stronger connection between the CS/NMNSs composite aerogel and *S. aureus* due to electrostatic interactions.^97^ Heba *et al.* studied the effects of nanoparticles on antibacterial properties by developing a chitosan/polyvinyl alcohol composite, modifying it with different weight percentages of NiO nanoparticles, and testing its antibacterial ability against two Gram-positive bacteria (*Staphylococcus aureus* and *Bacillus cereus*) and two Gram-negative bacteria (*Escherichia coli* and *Salmonella Typhimurium*). The results show that the modified NiO/chitosan/PVA aerogel has higher antibacterial activity than the unmodified chitosan/PVA aerogel because the presence of thiol groups (–SH) on the protein outer cell membrane attracts metallic ions, leading to damage to the bacterial membrane.^98^ Another study by Batista *et al.* investigated the antibacterial efficacy of alginate-chitosan aerogel fibres against *Staphylococcus aureus* and *Klebsiella pneumoniae* and compared them with Kaltostat's efficiency. Kaltostat is a calcium alginate-based wound dressing used for absorbing exudate and protecting wounds. The findings show that the alginate-chitosan aerogel fibres have a higher antibacterial activity than Kaltostat.^99^ Pan *et al.* developed an alginate/chitosan aerogel and assessed its antibacterial efficacy against *Staphylococcus aureus* and *Escherichia coli.* The aerogel demonstrated significant antibacterial efficacy, owing to its porous structure that enables direct interaction with bacteria. This interaction happens either *via* chemical bonding between the active cites within the pores and bacterial cells, or through the physical entrapment of bacteria within the porous matrix.^100^Table 3 illustrates different CS-based aerogels used for microorganism removal.

**Table 3:** Different CS-based aerogels for microorganisms' removal^a^

Aerogel	Microorganisms	Initial concentration CFU mL^−1^	Removal %	References
CS/NC/PA-Na	*S. aureus*	1 ×10^6^	78.5%	92
	*E. coli*		89.2%	
CS/CNFs	*S. aureus*	1.56 × 10^8^	99%	94
	*E. coli*	1.34 × 10^8^		
CS/NMNSs	*S. aureus*	1 × 10^8^	99%	95
	*E. coli*		97.52%	
CS/SBA-15	*S. aureus*	2.75 × 10^7^	100%	101
	*E. coli*	1.05 × 10^7^		
WPI/CS/CA/ε-PLH	*S. aureus*	—	80%	102
	*E. coli*			
	*Salmonella*			
	*Listeria monocytogenes*			
NADES/PVA/CS	*S. aureus*	10^6^	—	103
	*E. coli*			
Amino acid functionalized CS	*S. aureus*	—	∼100%	104
	*E. coli*			
	*V. parahaemolyticus*			
AG/CS	*S. aureus*	∼10^6^	22.03%	100
	*E. coli*		23.98%	
CS coated PCL/n-HA	*S. aureus*	3 × 10^5^	98%	105
	*E. coli*			
CSI	*S. aureus*	10^6^–10^7^	∼100%	106
	*E. coli*			

a CS: chitosan; NC: nano cellulose; PA-Na: sodium phytate; CNFs: cellulose nanofibers; NMNSs: amino-functionalized molybdenum disulfide; WPI: whey protein isolate; CA: citric acid; ε-PLH: ε-polylysine hydrochloride; *S. aureus*: *Staphylococcus aureus*; *E. coli*: *Escherichia coli*; PVA: polyvinyl alcohol; NADES: natural deep eutectic solvents; PCL: polycaprolactone; n-HA: nano-hydroxyapatite; AG: alginate; CSI: chitosan-itaconic acid.

### Microorganism removal mechanism

**Fig. 5:**
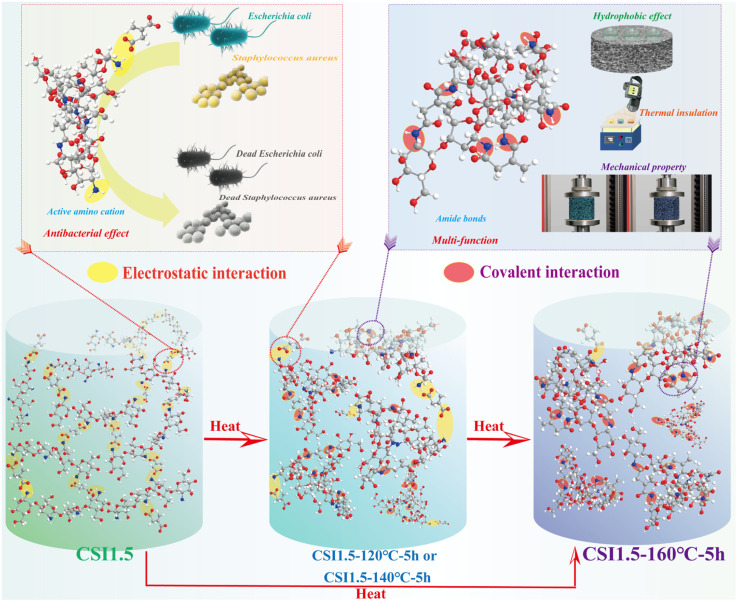
Antibacterial mechanism and multifunctionality of dual-network chitosan aerogel.^106^

The effective antibacterial properties of chitosan (CS) are due to the presence of protonated amino groups (–NH_3_^+^), which interact electrostatically with negatively charged bacterial cells. However, as mentioned before, CS has some drawbacks, such as low mechanical strength and hydrophilicity. For that reason, Yang *et al.* have developed a multifunctional dual-network chitosan/itaconic acid (CSI) aerogel with adaptability by freeze-drying and vacuum heat treatment procedures. Through the regulation of temperature and duration during the amidation reaction, the electrostatic interactions between chitosan (CS) and itaconic acid (IA) were properly transformed into amide bonds within frozen aerogel, with IA serving as an effective *in situ* cross-linking agent, resulting in CSI aerogels with varying electrostatic/covalent cross-linking ratios. The heat treatment and modification of the covalent cross-linking degree of CSI aerogel altered its microstructure and density, resulting in improved performance. This modification transformed the material from superhydrophilic to hydrophobic, thereby demonstrating enhanced stability and thermal transfer efficiency. Moreover, the development of chemically cross-linked sites (*i.e.*, amide bonds) and covalently cross-linked networks significantly improved the mechanical characteristics and water resistance (Fig. 5).^106^

### Removal of pharmaceuticals

The growth of population and developments in healthcare technology are driving the escalation of pharmaceutical drug production and consumption. Nevertheless, untreated hospital and pharmaceutical effluents, along with insufficient pharmaceutical waste management, have resulted in the discharge of medications into the environment. Pharmaceutical residues are enduring pollutants that present a considerable threat to human health, even at acceptable doses, due to their high toxicity and low biodegradability.^107^ Kim *et al.* synthesized a thermally-activated gelatin–chitosan aerogel and tested the removal efficiency toward ibuprofen (IBP) and naproxen (NPX) with an initial concentration of 3 mg L^−1^ and pH = 6. The results showed that the removal efficiency was 99.28% for IBP and 96.39% for NPX.^108^ In another study, Ravi and his co-workers prepared covalent organic polymers (COPs)/chitosan multifunctional aerogels and examined their removal ability against diclofenac sodium (DFS). The results showed that the aerogels exhibit effective (DFS) removal capabilities; nevertheless, their powdered nature renders them unsuitable for industrial use. For that, they developed an amine- and carboxyl group-rich aromatic COP (CCP-NH_2_)/chitosan aerogel and evaluated the adsorption ability to remove DFS from water. The findings indicate that the aerogel demonstrated a substantial surface area and mechanical strength, minimal density, remarkable stability in diverse solvents, and ease of processing into various forms. Furthermore, a substantial DFS adsorption capacity of 501 mg g^−1^.^109^ Natália *et al.* prepared hydrophobic CS@CNC-HTA beads aerogel to solve the hydrophilicity issue; they investigated the adsorption capacity for removing sildenafil citrate and cetylpyridinium chloride in water. The findings demonstrated that the removal rate of sildenafil citrate was 86 mg g^−1^ (73%), whereas it was 390 mg g^−1^ (90%) for cetylpyridinium chloride 52. Recently, Keli *et al.* synthesized crosslinked chitosan aerogel using bifunctional aldehydes (glutaraldehyde) as cross-linking agent and examined the adsorption removal of ibuprofen from water. The results showed that the cross-linked chitosan aerogel has excellent adsorption ability to remove ibuprofen with 596 mg g^−1^ adsorption capacity. The adsorption mechanism of ibuprofen onto chitosan aerogel is elucidated by the protonation of chitosan's amine groups (NH_3_^+^) under acidic conditions, resulting in a positively charged molecule. Ibuprofen includes carboxyl groups (–COOH) that can ionize, acquiring a negative charge (–COO^−^). Consequently, these attributes promote electrostatic interactions and hydrogen bonds between the protonated amino groups of chitosan and the ionised carboxyl groups of ibuprofen. Moreover, van der Waals interactions facilitate the adsorption of ibuprofen onto the porous surfaces of the aerogel, particularly in regions of near molecular contact.^110^ Balkis *et al.* synthesized chitosan/silica (CS/silica) aerogels characterized by high surface area and porosity and examined their adsorption efficacy for three pharmaceutical compounds at different silica weight ratios. The incorporation of silica markedly enhanced internal porosity and surface area, resulting in improved removal efficiency.^111^ Many studies used different CS-based aerogels to remove various types of pharmaceuticals, as shown in Table 4.

**Table 4:** Different CS-based aerogels for pharmaceuticals removal^a^

Aerogel	Pharmaceuticals	Initial concentration mg L^−1^	pH	Adsorption capacity mg g^−1^	Removal %	References
CGC-MOF	IBP	3	7	—	99.28%	108
	NPX				93.39%	
CCN-AG	DFS	30	4	501	∼98%	109
CS@CNC-HTA	SIL	100	6	86	73%	52
	CPC	100		390	90%	
CS	IBP	100	2	596	83%	110
Silica/CS	Carbamazepine	180.42	8.5	1610	96.8%	111
	IBP	201.92		1773	95.4%	
	Diclofenac	51.83		390	97.4%	
CS	RIF	30	7	66.91	—	112
	STM			11		
	IBP			24.21		
CS/ABC	KTP	50	4	130.29	—	113
ZIF-67/QGO/SB-CS	DOX	10	—	1300	—	114
	TC	400		1375		
	RFP	1000		100		
	MNZ	100		300		
	IBP	10		250		

a IBP: ibuprofen; NPX: naproxen; CGC: chitosan–Gelation-composite; MOF: metal organic framework; DFS: diclofenac sodium; CCN: covalent organic polymer-chitosan-aerogel; CNC: cellulose nanocrystals; HTA: hydrophobized tannic acid; SIL: sildenafil citrate; CPC: cetylpyridinium chloride; RIF: rifampicin; STM: streptomycin; ABC: activated biochar; KTP: ketoprofen; TC: tetracycline; DOX: doxorubicin hydrochloride; RFP: rifampicin; MNZ: metronidazole; ZIF: metal–organic framework; QGO: benzoquinone-functionalized GO; SB-CS: sulfobetaine-modified chitosan.

### Pharmaceuticals removal mechanism

**Fig. 6:**
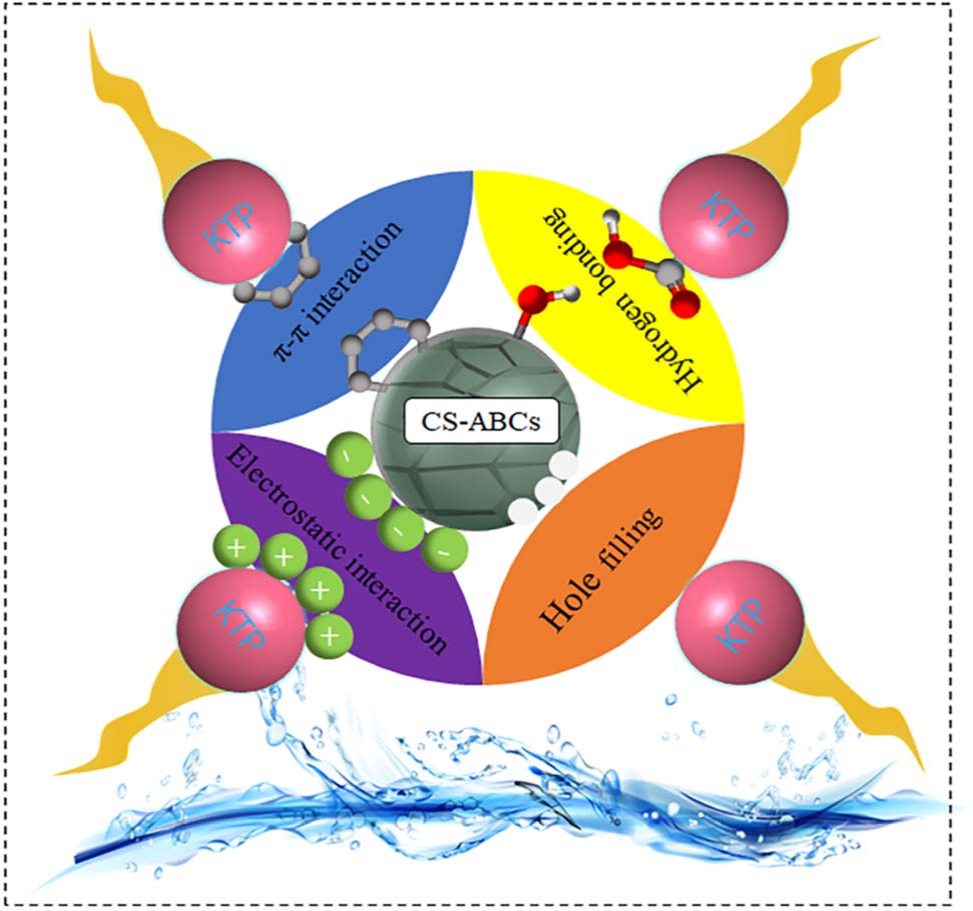
Possible adsorption mechanisms for KTP removal using CS-ABCs aerogel.^113^

Chen *et al.* prepared ultralight chitosan/activated biochar composite aerogel (CS-ABCs) and evaluated the adsorption capacity for ketoprofen (KTP) removal with a 50 mg L^−1^ initial concentration. They found that the maximum adsorption capacity under pH = 4 was 130.29 mg g^−1^. Fig. 6 illustrates four potential mechanisms for the sorption of KTP from aqueous solution onto CS-ABCs. (1) Hydrogen bonding occurred due to the abundant hydroxyl groups of CS-ABCs acting as hydrogen acceptors, while the carboxyl groups of KTP served as hydrogen donors. (2) π–π interactions were established between the graphitized structure of activated biochar in CS-ABCs and the aromatic structures present in KTP molecules. (3) Electrostatic interaction occurred as positively charged composite aerogel globules interacted with KTP anions in the solution. (4) Hole filling was facilitated by the developed 3D layered network structure within the composite aerogel globules, which featured suitable pore sizes (3.8 nm, 5.7 nm and 9.6 nm) and a porous activated biochar skeleton that supported the attachment of nanoscale KTP molecules (volume = 657.639 Å^3^).^113^

### Limitations and future aspects

Chitosan-based aerogels have garnered interest as sustainable and multifunctional materials for addressing diverse water pollutants, such as dyes, heavy metals, microbes, and pharmaceutical residues. Nevertheless, despite their potential, several significant restrictions persist. Their structural stability typically deteriorates in neutral and alkaline pH environments, adversely affecting both durability and reusability. A significant concern is that numerous studies conducted to date focus on single-pollutant systems in controlled batch environments, which may not accurately reflect the complexities of real wastewater streams. The development of aerogel formulations with improved chemical stability and the capability to target multiple contaminants is essential. Integrating chitosan aerogels into hybrid systems, such as membrane technologies or catalytic platforms, may improve their operational efficacy. Furthermore, incorporating studies on virus elimination could enhance their environmental and medicinal significance. The ultimate goal of future initiatives continues to be bridging the gap between successful laboratory experiments and real-world application.

## Conclusion

Recent advancements in application of chitosan-based aerogel adsorbents demonstrate their potential as environmentally friendly, efficient, and biodegradable materials for the removal of various contaminants, including dyes, heavy metals, microorganisms, and pharmaceutical waste ions, which are detrimental to aquatic ecosystems and living organisms. Furthermore, chitosan-based aerogel possesses a high number of –NH_2_ and –OH groups, acting as binding sites for the removal of these contaminant ions *via* electrostatic interactions, hydrogen bonding, or chelation. However, it exhibits some drawbacks, such as poor mechanical strength, dense structure, hydrophilicity, and lower porosity, which could affect the adsorption capacity. These issues have been solved using different techniques like blending with nanofillers, chemical and/or ionic cross-linkers, and designing composite aerogels. The modified chitosan aerogel exhibits remarkable adsorption capabilities toward different types of dyes, heavy metals, microorganisms, and pharmaceutical ions due to the abundance of –NH_2_ groups that exist on the aerogel surface. Many factors affect adsorption performance, among them pH. For instance, at acidic conditions, the –NH_2_ groups are protonated, making the aerogel positively charged, which binds with the negative charge of contaminant ions by electrostatic interaction. Meanwhile, in alkaline conditions, the –NH_2_ groups deprotonated, leading to the aerogel having a negative charge, causing it to bind electrostatically with the positive charge of contaminant ions. Also, the modified aerogel presented excellent antibacterial activity not only by adsorbing the microorganisms but even by eliminating them, making it a promising candidate in biomedical applications.

## Conflicts of interest

The authors declare no conflict of interest.

## Data Availability

No primary research results, software or code have been included and no new data were generated or analyzed as part of this review.
